# Supplementary material for: Automated, high-throughput in situ hybridization of sea urchin (Lytechinus pictus) embryos
Source: Development. 2025 Sep 22;152(18):dev204814. doi: 10.1242/dev.204814 (PMC12516323; doi:10.1242/dev.204814)
Supplement: Supplementary information [file develop-152-204814-s1.pdf]

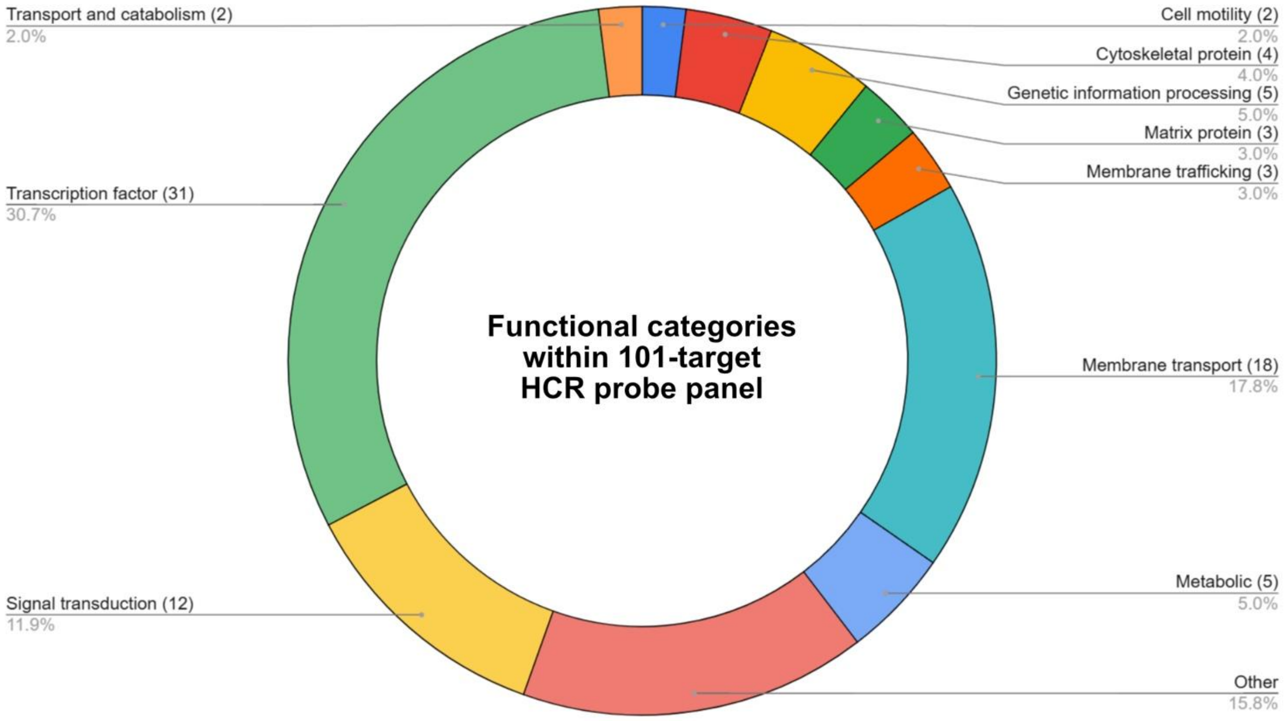

Fig. S1. Composition by gene functional category of the core HCR probe panel used in this study.

Development • Supplementary information

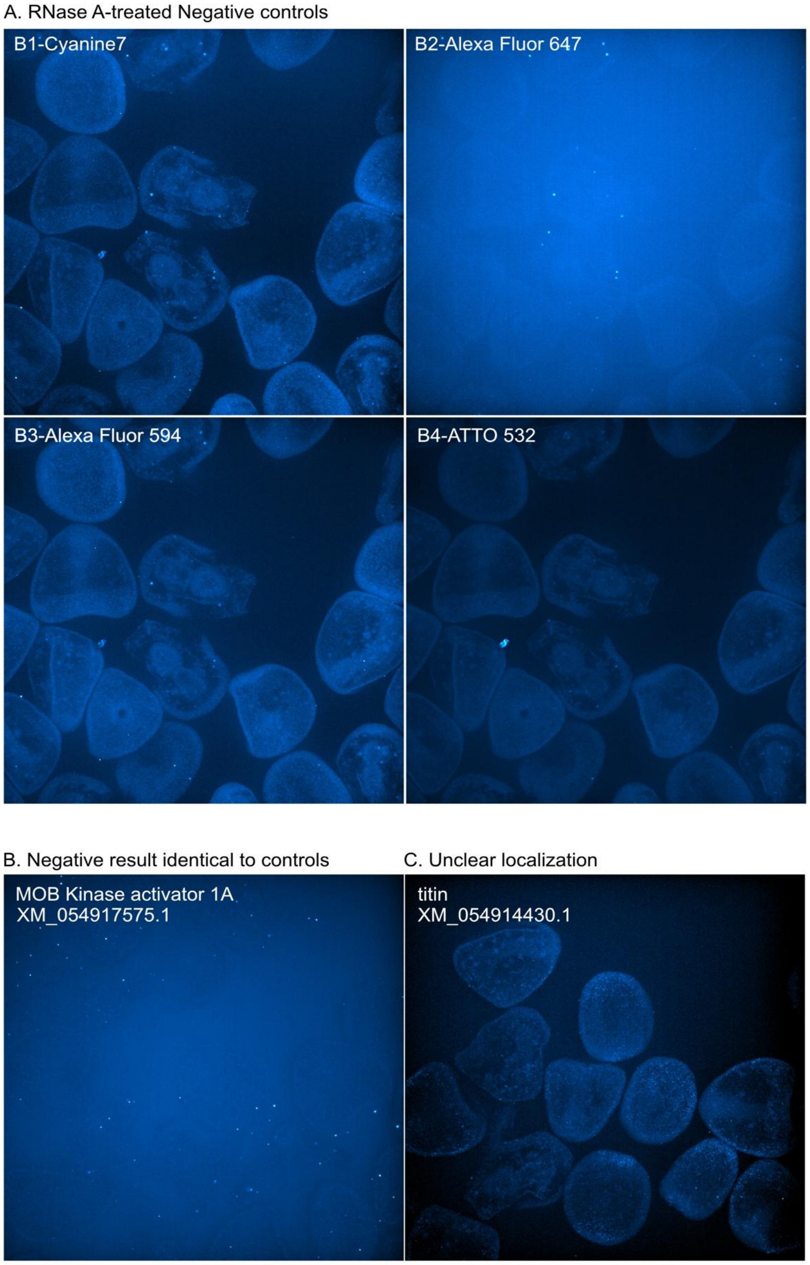

Fig. S2. RNAse A-treated negative control samples and genes which do not show clear localization.
A) RNAse-treated negative control samples show no specific localization for the positive control probes (B1-*msp130*, B2-*FoxG*, B3-*calmodulin*, and B4-*chordin*) applied to the sample. B) Example of a gene (*MOB kinase activator 1A*; XM_054917575.1) whose signal appears identical to negative controls. C) Example of a gene (*titin*; XM_054914430.1) where signal is present but the localization is- not clearly interpretable.

Development • Supplementary information

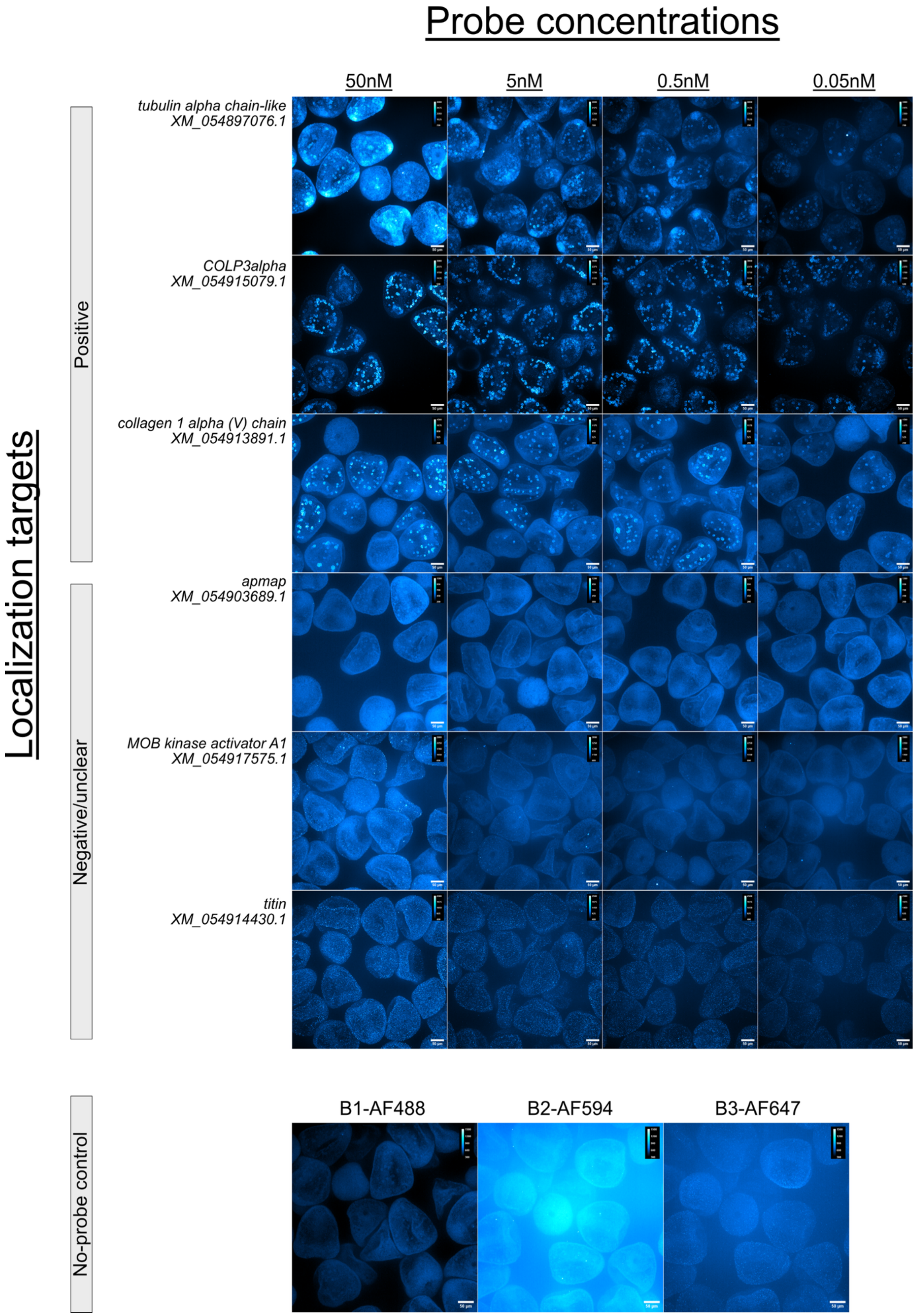

Fig. S3. HCR negative results do not appear to be a result of insufficient probe concentration.

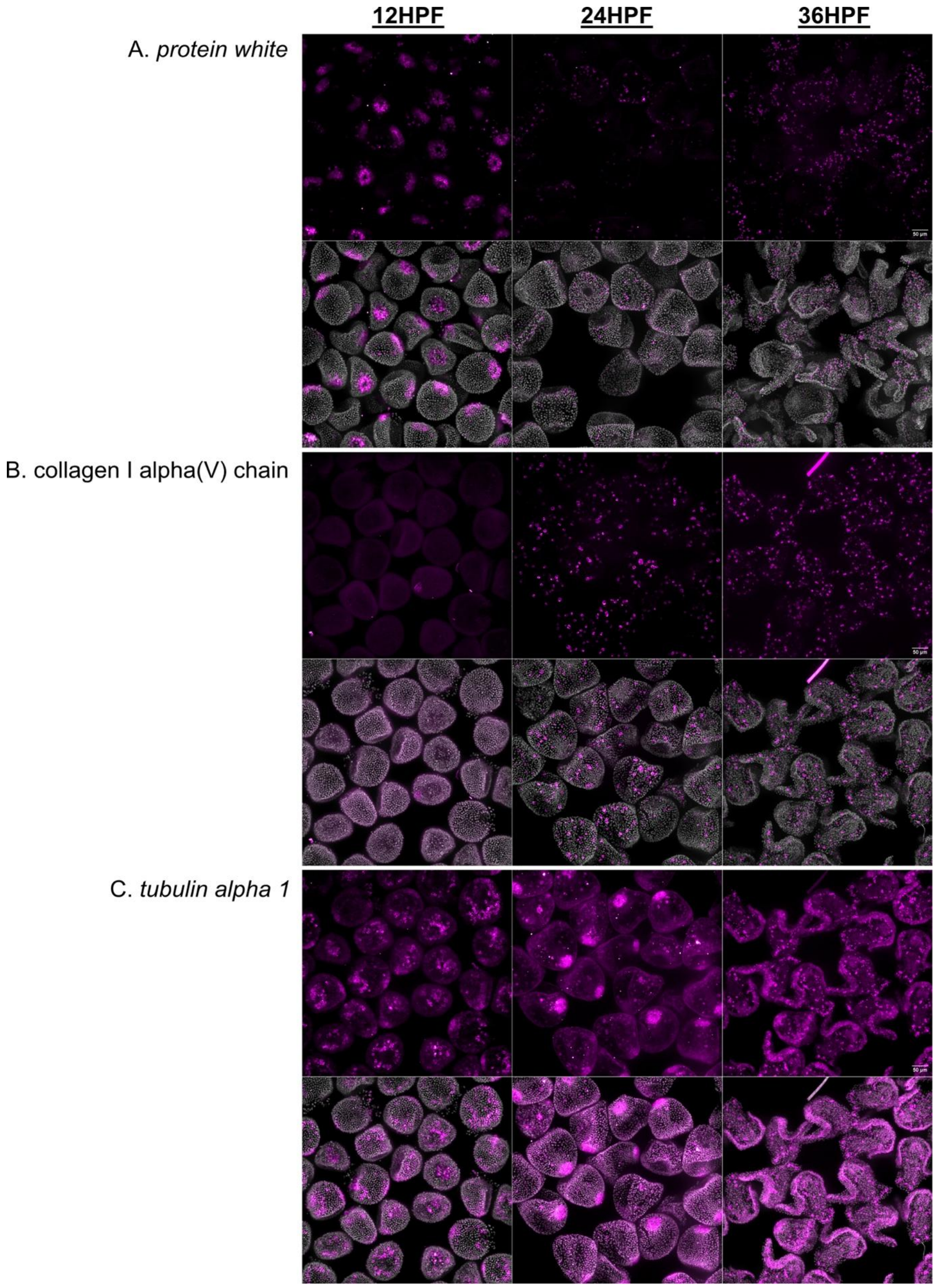

Fig. S4. Automated sample processing for HCR produces clear localization patterns across different developmental stages.

A) Localization of *protein white* (XM_054901325.1) in 12 hpf, 24 hpf, and 36 hpf embryos. In 12 hpf embryos, *protein white* is expressed in the NSM. In 24 and 36 hpf embryos, *protein white* is expressed in pigment cells. B) Localization of *collagen I alpha(V) chain* (XM_054913891.1) in 12 hpf, 24 hpf, and 36 hpf embryos. Expression is absent in 12 hpf embryos. In 24 and 36 hpf embryos, *collagen I alpha(V) chain* is expressed in mesodermal cells. C) Localization of *tubulin alpha 1* (XM_054897478.2) in 12 hpf, 24 hpf, and 36 hpf emrbyos. Expression of *tubulin alpha 1* is ubiquitous but highly enriched in mesenchymal cells across all stages. In 24 hpf embryos, expression is enriched in the apical organ, and in 36 hpf embryos, expression is enriched in the ciliary band.

Development • Supplementary information

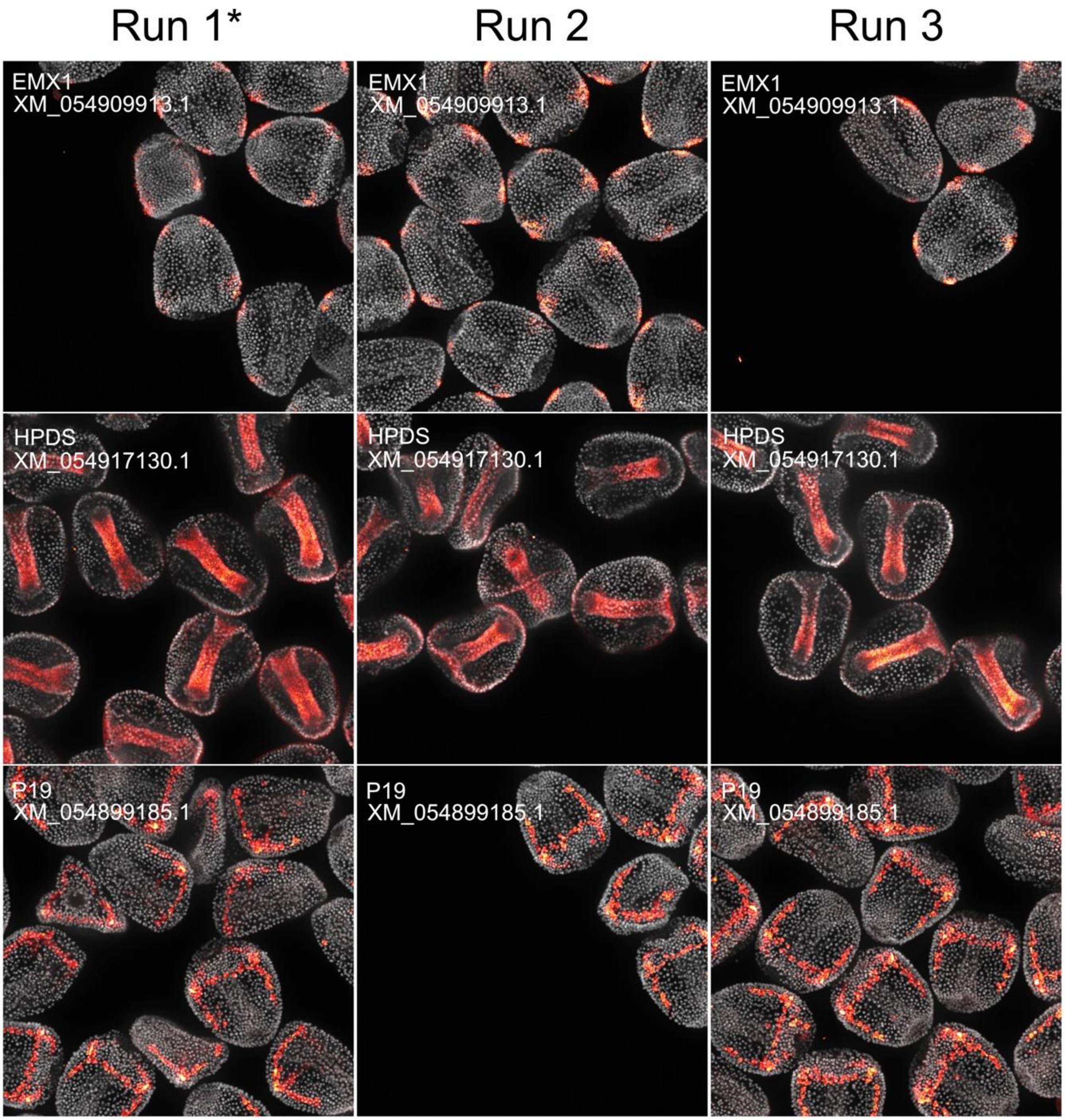

Fig. S5. Localization of genes is consistent across different runs of HT-HCR.
Expression for *EMX1, HPDS,* and *P19* are consistent across three separate runs of HT-HCR. Asterisk (*) denotes images of genes which exist in another figure in this publication from the same run (Fig. 2).

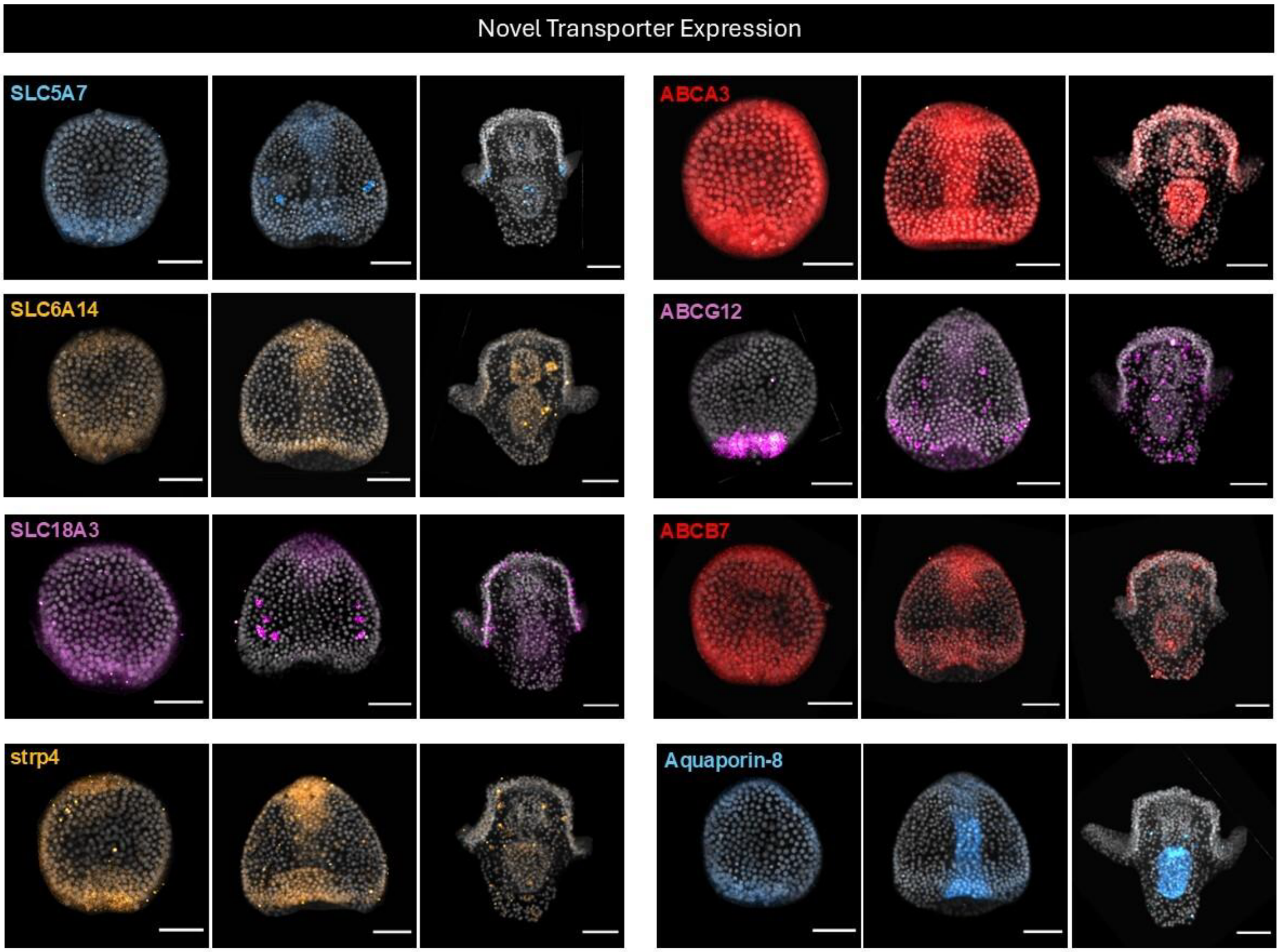

Fig. S6. Individual embryo samples can be isolated from larger full-scale images.

Table S1. Genes which constitute 101 positive localizations in this study. "Ubi" = ubiquitous

Accession	Gene Name	# Probe Pairs	Hairpin	Channel	12 hpf localization	24 hpf localization	36 hpf localization
XM_054915350.2	14-3-3 protein 2	12	b4	Cy7	Ubi	Ubi	Ubi
XM_054908194.1	ABCA1	15	b4	Cy7	NSM	Ectoderm	NA
XM_054906925.1	ABCA3	15	b3	Alexa Fluor 647	Ubi	Ubi	Endoderm
XM_054906809.1	ABCA5	15	b1	ATTO 532	Ectoderm	Ectoderm	Ectoderm
XM_054911547.1	ABCB1	15	b3	Alexa Fluor 647	Ubi	Ubi	Ubi
XM_064109812.1	ABCB4	15	b1	ATTO 532	NA	Ubi	Endoderm
XM_054897890.1	ABCB7	15	b2	Alexa Fluor 594	Ubi	Ubi	Mesoderm
XM_054906725.1	ABCC1	15	b2	Alexa Fluor 594	Ubi	Ubi	NA
XM_054911957.1	ABCG12	15	b2	Alexa Fluor 594	NSM	Mesoderm	Mesoderm
XM_054917858.1	ABCG2	15	b3	Alexa Fluor 647	Ubi	Endoderm	Endoderm
XM_054914077.1	Actin, cytoskeletal 1A	15	b4	Cy7	NA	Mesoderm, Ectoderm	Ubi, Mesoderm
XM_054906503.1	ankyrin repeat domain-containing protein 55	15	b4	Cy7	Ectoderm	Apical	NA
XM_054901620.1	APOBEC1 complementation factor (a1cf)	9	b3	Alexa Fluor 647	NA	Endoderm	Endoderm
XM_054907444.1	aquaporin-8	10	b4	Cy7	NA	Endoderm	Endoderm
XM_064111875.1	axotactin	15	b2	Alexa Fluor 594	Mesoderm	Mesoderm	Mesoderm
XM_054900056.1	beta-catenin	10	b4	Cy7	Ubi	Ubi	Ubi
XM_054900733.1	betaine-homocysteine S-methyltransferase 1	10	b3	Alexa Fluor 647	NA	Endoderm	Endoderm
XM_054897283.1	blimp1a	10	b3	Alexa Fluor 647	NA	Endoderm	Endoderm
XM_054917286.1	bmp2	10	b3	Alexa Fluor 647	NA	Endoderm/ Mesoderm	Endoderm
XM_054900839.1	calmodulin	4	b4	Cy7	NA	Endoderm	Endoderm

Development • Supplementary information

XM_054896288.1	caveolin-3	8	b3	Alexa Fluor 647	NA	Endoderm/Ectoderm	Endoderm/Ectoderm
XM_054906303.1	CD151 antigen	10	b2	Alexa Fluor 594	NA	Endoderm	Endoderm
XM_054912384.1	chordin	10	b1	ATTO 532	Ectoderm	Ectoderm	Ectoderm
XM_054913891.1	collagen alpha-1(I/V) chain	10	b3	Alexa Fluor 647	NA	Mesoderm	Mesoderm
XM_054915079.1	collagen alpha-3(IV) chain/COLP3alpha	10	b1	ATTO 532	NSM	Mesoderm	Mesoderm
XM_054895445.2	COLP1alpha	15	b4	Cy7	NA	Mesoderm	Mesoderm
XM_054892573.1	cyclophilin-1	9	b4	Cy7	NA	Mesoderm	Mesoderm
XM_054914495.1	D-dopachrome decarboxylase	5	b1	ATTO 532	NSM	Mesoderm	Mesoderm
XM_054911692.1	dickkopf WNT signaling pathway inhibitor 3-like	10	b3	Alexa Fluor 647	Ectoderm	Ectoderm	Ectoderm
XM_054899987.1	dri	10	b2	Alexa Fluor 594	NA	Ectoderm	NA
XM_054908153.1	ELAV3	15	b3	Alexa Fluor 647	Mesoderm	Mesoderm	Mesoderm
XM_054902560.1	elongation factor 1 gamma	15	b2	Alexa Fluor 594	Ubi	Ubi	Endoderm/Mesoderm
XM_054903584.1	EMAP77	15	b1	ATTO 532	Ectoderm	Ectoderm	Ectoderm
XM_054919060.1	Endo16	10	b4	Cy7	Endoderm	Endoderm	Endoderm
XM_054904347.1	erg	10	b3	Alexa Fluor 647	Mesoderm	Mesoderm	NA
XM_054905949.1	ese	10	b2	Alexa Fluor 594	NSM	Ectoderm	Ectoderm
XM_054916386.1	eyes absent	10	b2	Alexa Fluor 594	NA	NA	Mesoderm
XM_064102407.1	fatty acid-binding protein type 3	7	b1	ATTO 532	NA	Endoderm	Endoderm
XM_054895154.1	fezf2	10	b3	Alexa Fluor 647	NA	Ectoderm	Ectoderm
XM_054914245.1	ficolin-3	10	b2	Alexa Fluor 594	Mesoderm	Mesoderm	Mesoderm
XM_054903096.1	FoxA	10	b4	Cy7	Endoderm	Endoderm	Endoderm
XM_054892407.1	FoxC	10	b2	Alexa Fluor 594	NA	Mesoderm	Mesoderm
XM_054903002.1	FoxG	10	b2	Alexa Fluor 594	NA	Ectoderm	NA

XM_054906495.1	FoxQ2	10	b1	ATTO 532	Ectoderm	Apical	NA
XM_054897154.1	frizzled-4	15	b2	Alexa Fluor 594	NA	Endoderm/Mesoderm	Endoderm/Mesoderm
XM_054897832.1	frizzled-5	10	b4	Cy7	Endoderm	Endoderm	Endoderm
XM_054916700.2	gcm	10	b1	ATTO 532	NSM	Mesoderm	Mesoderm
XM_054917843.1	gelsolin-2	15	b4	Cy7	NA	Mesoderm	Mesoderm
XM_054895438.1	Hbox7	5	b2	Alexa Fluor 594	Endoderm	Endoderm	Endoderm
XM_054917130.1	hematopoietic prostaglandin D synthase (HPDS)	5	b4	Cy7	NA	Endoderm	Endoderm
XM_054909913.1	homeobox protein EMX1	10	b1	ATTO 532	Ectoderm	Ectoderm	Ectoderm
XM_054900719.1	homeobrain	10	b2	Alexa Fluor 594	Ectoderm	Ectoderm	Ectoderm
XM_054902120.1	left-right determination factor 2	10	b1	ATTO 532	Ectoderm	Ectoderm	Ectoderm
XM_054895022.1	ManR	15	b4	Cy7	NA	NA	Endoderm
XM_054900255.1	matrix metalloproteinase-2	10	b2	Alexa Fluor 594	NSM	Mesoderm	Mesoderm
XM_054896825.1	metallothionein-A	3	b1	ATTO 532	NA/mesoderm	NA	Mesoderm
XM_054912810.1	msp130	10	b1	ATTO 532	Mesoderm	Mesoderm	Mesoderm
XM_054893990.1	mucin-17	15	b3	Alexa Fluor 647	NSM	Mesoderm	Mesoderm
XM_054899132.1	mucin-2	15	b4	Cy7	Ectoderm	Ectoderm	Ectoderm
XM_054917449.1	musashi1	13	b1	ATTO 532	NA	Endoderm	Endoderm
XM_064102363.1	AHNAK	15	b4	Cy7	Ectoderm	Ectoderm	Ectoderm
XM_054892815.1	odd-skipped-related 1	10	b3	Alexa Fluor 647	Ectoderm/Endoderm	Endoderm	Endoderm
XM_054901727.1	onecut2	10	b2	Alexa Fluor 594	Ectoderm	Ectoderm	Ectoderm
XM_054899185.1	P19	5	b3	Alexa Fluor 647	Mesoderm	Mesoderm	Mesoderm
XM_054908715.1	patched homolog 1-like	15	b3	Alexa Fluor 647	Ectoderm	Ectoderm	Mesoderm
XM_054917528.1	pax2/5/8	9	b3	Alexa Fluor 647	Ectoderm	Ectoderm	Ectoderm
XM_054896981.1	POU domain, class 3, transcription factor	10	b1	ATTO 532	NA	Endoderm/Ectoderm	Endoderm/Ectoderm
XM_054902058.1	prospero homeobox protein 1	10	b3	Alexa Fluor 647	NSM	Mesoderm/Ectoderm	Ectoderm

Development • Supplementary information

XM_054901731.1	protein PB18E9.04c	15	b2	Alexa Fluor 594	Ectoderm	Ectoderm	Ectoderm
XM_054911221.1	protein rolling stone	11	b1	ATTO 532	NA	Mesoderm	Mesoderm
XM_054901325.1	protein_white	15	b2	Alexa Fluor 594	NSM	Mesoderm	Mesoderm
XM_054899164.1	protocadherin Fat 1-like	15	b4	Cy7	Ectoderm	Ectoderm	Ectoderm
XM_054912035.1	ptfa1	10	b1	ATTO 532	NA	Endoderm/Ectoderm	Endoderm
XM_054915559.1	quinone oxidoreductase	10	b1	ATTO 532	NSM	Mesoderm	Mesoderm
XM_054913367.1	scratch	10	b4	Cy7	NA	Mesoderm	Mesoderm
XM_064095033.1	short transient receptor potential channel 4	15	b3	Alexa Fluor 647	NSM	Mesoderm	Mesoderm
XM_054902743.1	six1	10	b2	Alexa Fluor 594	NA	Mesoderm	Mesoderm
XM_054902744.1	six3/6	9	b4	Cy7	NA	Mesoderm	Mesoderm
XM_054900099.1	SLC13A5	15	b1	ATTO 532	NA	Mesoderm	Mesoderm
XM_054910749.1	SLC18A2	15	b1	ATTO 532	NA	Mesoderm	Mesoderm
XM_054918215.1	SLC18A3	15	b4	Cy7	NA	Mesoderm	Ectoderm
XM_054913204.1	SLC2A1	15	b1	ATTO 532	NA	NA	Ubi/Endomesoderm
XM_054909489.1	SLC5A7	15	b2	Alexa Fluor 594	NA	Mesoderm	Mesoderm
XM_054913933.1	SLC6A14	10	b1	ATTO 532	Ubi	Ubi	Mesoderm
XM_054919026.1	SM34	10	b4	Cy7	Mesoderm	Mesoderm	Mesoderm
XM_054914671.1	Sox-3-B	10	b2	Alexa Fluor 594	Ubi	Ubi	Ubi
XM_054902877.1	Spec3	6	b2	Alexa Fluor 594	Ectoderm	Ectoderm	Ectoderm
XM_054918853.2	synaptotagmin-1	13	b3	Alexa Fluor 647	NA	Mesoderm	Ectoderm/Mesoderm
XM_054898009.1	T-box brain transcription factor 1	10	b2	Alexa Fluor 594	Mesoderm	Mesoderm	NA
XM_054897143.1	T-box transcription factor T (brachyury)	10	b1	ATTO 532	Endoderm	Endoderm/Ectoderm	NA
XM_054902833.1	thyroid transcription factor 1	10	b4	Cy7	Ectoderm	Ectoderm	Ectoderm
XM_054898855.1	thyrotroph embryonic factor-like	11	b3	Alexa Fluor 647	Ubi	Ectoderm/Mesoderm	Ectoderm/Mesoderm
XM_054910758.1	transcription factor coe	10	b3	Alexa Fluor 647	NA	Ectoderm	Ectoderm

Development • Supplementary information

XM_054906577.1	transcription factor SOX-9	10	b1	ATTO 532	NA	Mesoderm	Mesoderm
XM_054905379.1	troponin I	10	b1	ATTO 532	NA	Endoderm	Endoderm
XM_054897076.1	tubulin alpha chain-like	15	b1	ATTO 532	Ectoderm/Mesoderm	Ectoderm/Mesoderm	Ectoderm/Mesoderm
XM_054897478.2	tubulin alpha-1 chain	15	b4	Cy7	Ectoderm/Mesoderm	Ectoderm/Mesoderm	Ectoderm/Mesoderm
XM_054902965.1	univin	10	b2	Alexa Fluor 594	Ectoderm	Ectoderm	Ectoderm
XM_054917943.1	wnt8	10	b3	Alexa Fluor 647	Ectoderm	Ectoderm	NA
XM_054895436.1	XHOX-3	10	b2	Alexa Fluor 594	Ectoderm	Ectoderm	NA
XM_054897132.1	zinc finger C4H2 domain contaning protein	9	b2	Alexa Fluor 594	Ectoderm	Mesoderm	Mesoderm

Table S2. Genes which failed to produce clearly interpretable localization.

Accession	Gene Name	# Probe Pairs	Hairpin	Channel
XM_054913519.1	abca2	15	b1	ATTO 532
XM_054916213.1	abca4	15	b2	Alexa Fluor 594
XM_054902369.1	abcb10	15	b2	Alexa Fluor 594
XM_054911894.1	abcb6	15	b4	Cy7
XM_054893952.1	abcb9	15	b3	Alexa Fluor 647
XM_054893102.1	abcc10a	15	b1	ATTO 532
XM_054902028.1	abcc10b	15	b1	ATTO 532
XM_054913988.1	abcc4_X1_2*	15	b1	ATTO 532
XM_054915178.1	abcc4_X1*	15	b3	Alexa Fluor 647
XM_054915179.1	abcc4_X2*	15	b4	Cy7
XM_054915180.1	abcc4_X3*	15	b2	Alexa Fluor 594
XM_054915181.1	abcc4_X4*	15	b3	Alexa Fluor 647
XM_054896220.1	ABCC9	15	b2	Alexa Fluor 594

Development • Supplementary information

XM_054903689.1	adipocyte plasma membrane-associated protein	15	b3	Alexa Fluor 647
XM_054913502.1	allograft inflammatory factor 1-like	6	b2	Alexa Fluor 594
XM_054900595.1	ameboid myosin I	15	b2	Alexa Fluor 594
XM_054898670.1	aryl_hydricarbon_receptor_nuclear_translocator_homolog	15	b1	ATTO 532
XM_054906884.1	brain-specific angiogenesis inhibitor 1-associated protein 2	15	b1	ATTO 532
XM_054915270.1	BRCA2-interacting_transcriptional_repressor_EMSY-like	15	b3	Alexa Fluor 647
XM_054900846.1	caspase_3_like*	15	b1	ATTO 532
XM_054897466.1	caspase_3_like*	15	b2	Alexa Fluor 594
XM_054893801.1	caspase_6_like	15	b4	Cy7
XM_054919029.1	catalase-like	15	b4	Cy7
XM_054905955.1	CD9 antigen	9	b2	Alexa Fluor 594
XM_054892002.1	coronin-1B	13	b4	Cy7
XM_054904859.1	cubilin-like	15	b3	Alexa Fluor 647
XM_054896836.1	cytochrome_P450_1A1_like*	15	b1	ATTO 532
XM_054904951.1	cytochrome_P450_1A1_like*	15	b3	Alexa Fluor 647
XM_054906659.1	cytochrome_P450_1A1_like*	15	b4	Cy7
XM_054906733.1	DNA ligase 1	10	b2	Alexa Fluor 594
XM_054910483.1	drebrin-like protein A	12	b2	Alexa Fluor 594
XM_054893174.1	hnf1a	10	b4	Cy7
XM_041598698.1	hnf4	15	b3	Alexa Fluor 647

Development • Supplementary information

XM_054901951.1	homeobox protein homothorax-like	4	b1	ATTO 532
XM_054907821.1	homeobox protein Unc−4	10	b4	Cy7
XM_054894536.1	hox1	6	b3	Alexa Fluor 647
XM_054896340.1	hox9	10	b3	Alexa Fluor 647
XM_054906011.1	hsp70-binding_protein_1_like	14	b2	Alexa Fluor 594
XM_054919221.1	hsp90_co_chaperone_cdc_37_like	15	b3	Alexa Fluor 647
XM_054914601.1	hyalin-like	15	b2	Alexa Fluor 594
XM_054899136.1	Kibra-like	15	b4	Cy7
XM_054907292.1	lethal(2) giant larvae protein homolog 1-like	3	b4	Cy7
XM_054897344.1	macoilin	13	b1	ATTO 532
XM_054908910.1	macrophage migration inhibitory factor	6	b4	Cy7
XM_054897977.1	metal_regulatory_transcription_factor_1_like	9	b3	Alexa Fluor 647
XM_054913983.1	metal_response_element_binding_transcription_factor_2_like	15	b4	Cy7
XM_054906133.1	microtubule-associated_protein_futsch-like	15	b1	ATTO 532
XM_054917575.1	MOB kinase activator 1A-like	10	b2	Alexa Fluor 594
XM_054906288.1	MOB kinase activator 2-like	11	b3	Alexa Fluor 647
XM_054897489.1	mtf1	15	b3	Alexa Fluor 647
XM_054899059.1	mucin-12	15	b2	Alexa Fluor 594
XM_054905346.1	mucin-17_like*	15	b3	Alexa Fluor 647
XM_054893754.1	mucin-17_like*	15	b3	Alexa Fluor 647
XM_054909363.1	mucin-19	15	b4	Cy7

XM_054895498.1	mucin-2_like*	15	b2	Alexa Fluor 594
XM_054905746.1	mucin-2_like*	15	b2	Alexa Fluor 594
XM_054892398.1	mucin-2_like*	15	b4	Cy7
XM_054893604.1	mucin-5ac	15	b1	ATTO 532
XM_054905261.1	mucinl	15	b2	Alexa Fluor 594
XM_054919698.1	neuronal acetylcholine receptor subunit alpha-10	14	b2	Alexa Fluor 594
XM_054912738.1	neuronal acetylcholine receptor subunit alpha-2	15	b4	Cy7
XM_054915363.1	neurotrypsin	10	b4	Cy7
XM_054899223.1	octopamine receptor	10	b1	ATTO 532
XM_054901330.1	plastin-3	15	b4	Cy7
XM_054902250.1	protein salvador homolog 1-like	15	b3	Alexa Fluor 647
XM_054904308.1	remodeling and spacing factor 1	13	b4	Cy7
XM_054899962.1	retina and anterior neural fold homeobox protein	9	b1	ATTO 532
XM_054904870.1	roundabout homolog 1	15	b1	ATTO 532
XM_054899015.1	secretory carrier-associated membrane protein 1	14	b1	ATTO 532
XM_054916437.1	Serine/threonine-protein kinase 3-like (hippo)	14	b4	Cy7
XM_054899570.1	Serine/threonine-protein kinase 4-like (hippo)	15	b2	Alexa Fluor 594

Development • Supplementary information

XM_054896280.1	serine/threonine-protein kinase A-Raf-like	15	b1	ATTO 532
XM_054897360.1	serine/threonine-protein kinase LATS2-like	15	b1	ATTO 532
XM_054896684.1	slc22a4	15	b3	Alexa Fluor 647
XM_054913357.1	slc31a1	15	b3	Alexa Fluor 647
XM_054898789.1	smad1/5/8	10	b3	Alexa Fluor 647
XM_054906962.1	SOCS2	8	b4	Cy7
XM_054903022.1	spastin	7	b3	Alexa Fluor 647
XM_054900002.1	sprouty homolog 3-like	12	b3	Alexa Fluor 647
XM_054907292.1	sushi	15	b4	Cy7
XM_054919067.1	synaptotagmin-7	14	b4	Cy7
XM_054895192.1	TAZ	9	b3	Alexa Fluor 647
XM_054918745.1	TEF1-like	15	b1	ATTO 532
XM_054893323.1	tektin1	15	b4	Cy7
XM_054914430.1	titin	15	b1	ATTO 532
XM_054904575.1	tyrosine-protein kinase JAK2	13	b3	Alexa Fluor 647
XM_054919211.1	tyrosine-protein_phosphatase_10D-like	15	b2	Alexa Fluor 594
XM_054894772.1	wnt16	10	b1	ATTO 532
XM_054915720.1	YAP1-A	7	b1	ATTO 532
XM_054914833.1	YAP1-B	6	b2	Alexa Fluor 594
XM_054909721.1	z60/egr	15	b4	Cy7
